# New In Vitro Cellular Model for Molecular Studies of Retinitis Pigmentosa

**DOI:** 10.3390/ijms22126440

**Published:** 2021-06-16

**Authors:** Li Huang, Meltem Kutluer, Elisa Adani, Antonella Comitato, Valeria Marigo

**Affiliations:** 1 Department of Life Sciences, University of Modena and Reggio Emilia, Via G. Campi 287, 41125 Modena, Italy; 251360@studenti.unimore.it (L.H.); meltem.kutluer@unimore.it (M.K.); elisa.adani@unimore.it (E.A.); antonella.comitato@unimore.it (A.C.); 2 Center for Neuroscience and Neurotechnology, Via Campi 287, 41125 Modena, Italy

**Keywords:** 661W, rod photoreceptor, neuroprotection, retinal degeneration

## Abstract

Retinitis pigmentosa (RP) is an inherited form of retinal degeneration characterized by primary rod photoreceptor cell death followed by cone loss. Mutations in several genes linked to the disease cause increased levels of cyclic guanosine monophosphate (cGMP) and calcium ion influxes. The purpose of this project was to develop a new in vitro photoreceptor degeneration model for molecular studies of RP. 661W cells were genetically modified to stably express the neural retina leucine zipper (NRL) transcription factor. One clone (661W-A11) was selected based on the expression of *Nrl* target genes. 661W-A11 showed a significant increase in expression of rod-specific genes but not of cone-specific genes, compared with 661W cells. Zaprinast was used to inhibit phosphodiesterase 6 (PDE6) activity to mimic photoreceptor degeneration in vitro. The activation of cell death pathways resulting from PDE6 inhibition was confirmed by detection of decreased viability and increased intracellular cGMP and calcium, as well as activation of protein kinase G (PKG) and calpains. In this new in vitro system, we validated the effects of previously published neuroprotective drugs. The 661W-A11 cells may serve as a new model for molecular studies of RP and for high-throughput drug screening.

## 1. Introduction

Retinitis pigmentosa (RP) is one form of inherited retinal degenerative disease (IRD) causing incurable blindness. It is characterized by the loss of retinal photoreceptor cells, the neurons specialized in converting light stimuli into electrical signals. Two types of photoreceptors are present in the human retina: rod photoreceptors expressing rhodopsin, which is very sensitive to photons, and cone photoreceptors, which express opsins for color vision and sight in daylight. The characteristics of photoreceptors make them unique, specialized cells in the human body [1]. RP is characterized by the primary loss of rods, followed by cone cell death. Inherited mutations in over 70 different genes are linked to RP and these genes are often expressed only in rod photoreceptors [2]. While a high number of different mutations can be detrimental to rod photoreceptors, some common molecular events characterize the progression of photoreceptor cell death and among these is the increase in cyclic guanosine monophosphate (cGMP) levels and in calcium influx [3,4,5,6]. Therapeutic approaches for the preservation of diseased photoreceptor cells face several challenges such as the high genetic heterogeneity [2], the impossibility of collecting biopsies from RP patients, the presence of a blood–retina barrier that hinders drug delivery [7] and the lack of an appropriate in vitro model for drug screening. This last issue relates to the fact that photoreceptor cells are postmitotic neurons and cannot regenerate themselves; thus, cell lines cannot be generated from adult photoreceptor cells. All these problems make the study of rod photoreceptors that undergo cell death in RP demanding.

Several retina cell lines exist for the study of inherited eye diseases [8,9,10,11]. However, photoreceptor cell lines are quite limited; examples are the human cell lines WERI-Rb1 and Y-79 [12,13,14]. The origin of these cell lines is retinoblastoma tumors. They are highly transformed with limited characteristics of rod photoreceptors, while expressing some rod-specific genes [15]. Procedures for in vitro differentiation of retinal stem cells into photoreceptors have been developed and have demonstrated that these cells are suitable for drug testing [16,17,18]. The drawback of this system is the high variability of primary cells that need to be isolated from murine eyes. 661W is the cell line mostly used by researchers working on the retina. This cell line was derived from an immortalized photoreceptor progenitor originating from retinal tumors in transgenic mice. The transgenic mice expressed simian virus 40 (SV40) T antigen under the transcriptional control of the human interphotoreceptor retinol-binding protein (*IRBP*) promoter, a gene expressed specifically in rod and cone photoreceptors [19]. The 661W cell line is considered a good cell model for the study of photoreceptor diseases because it expresses genes of photoreceptor precursors, with expression of some cone photoreceptor genes [20,21]. The expression of rod precursor genes, the growth as a monolayer and the possibility of inducing differentiation with specific treatments and to genetically modify these cells are characteristics that make this cell line a promising model for RP [22,23]. Nevertheless, a cell line with higher expression of rod photoreceptor-specific genes is necessary for the development of high-throughput in vitro drug screening systems for RP.

The regulation of gene expression by specific transcription factors during retinal histogenesis allows the progressive differentiation of the specific retinal cell types composing the retina [24]. One of the master genes during rod photoreceptor differentiation is *Nrl* (*neural retina-specific leucine*), which encodes the basic motif–leucine zipper (bZIP) transcription factor regulating rod-specific genes [25,26]. NRL, together with other two transcription factors, CRX (cone–rod homeobox protein) and NR2E3 (nuclear receptor subfamily 2, Group E, Member 3), acts as an activator for rod photoreceptor-specific genes [27,28,29]. The requirement of NRL for rod-specific differentiation was demonstrated by the cone-like features of photoreceptors that lack the *Nrl* gene and transformation of cones into rods upon *Nrl* misexpression in transgenic mice [30,31,32]. 

In this study, we developed a new in vitro model from 661W cells for the study of RP based on zaprinast treatment of rod-like cells. To induce the expression of rod-specific genes, 661W cells were genetically modified to stably express the NRL transcription factor. We present the molecular characterization of this new cell line and the validation of these cells as a new model for the study of retinal degeneration by mimicking the disease upon treatment with a phosphodiesterase 6 (PDE6) inhibitor, which raises intracellular cGMP. The validity of the cell line for high-throughput screening was confirmed by the effects of previously published neuroprotective drugs.

## 2. Results

### 2.1. Generation and Selection of One 661W Clone Expressing Nrl

**Figure 1:**
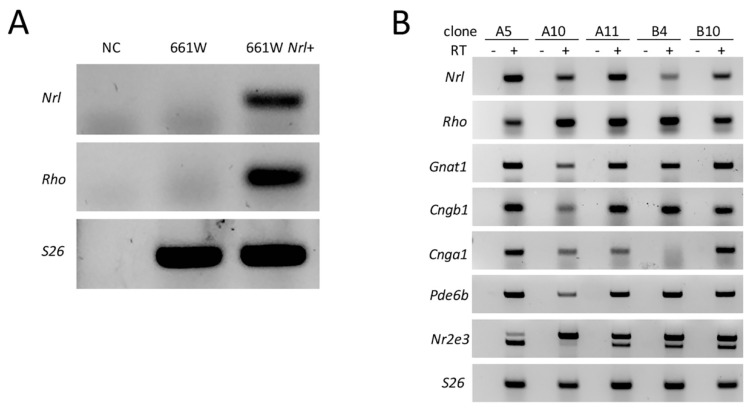
Generation and selection of *Nrl*-expressing 661W cells. (**A**) RT-PCR analysis of *Nrl* and *Rho* mRNA on 661W and 661W *Nrl*+ cells (bulk of retroviral transduced cells). *Ribosomal protein gene RPS26* (*S26)* was analyzed as a reference gene. Expression of *Nrl* and *Rho* was confirmed in 661W transduced cells but not 661W cells. NC: negative control without cDNA (retrotranscription −). (**B**) Five clones showed differential expression of rod-specific genes when analyzed by RT-PCR (RT - or +: retrotranscription − or +). *S26* was analyzed as a reference gene. The primers for *Nr2e3* also amplified a transcript variant with an intron inclusion reported in IMAGE:5357172. For *Nr2e3*, we evaluated only the lower molecular weight band corresponding to the spliced mRNA.

Murine *Nrl* cDNA was cloned in the pLXSN vector, and a replication-incompetent retrovirus was produced and used for transduction of 661W cells. Transduced cells were selected based on their neomycin resistance and analyzed by PCR for expression of *Nrl* and *Rhodopsin* (*Rho*) genes (Figure 1A). After selecting single-cell clones by limiting dilution, *Nrl* expression was assessed on RNA extracted from each cell clone by RT-PCR. Among these, we selected clones in which at least one known *Nrl* target was upregulated. Five clones out of 11 were selected, and we confirmed the expression of the *Nrl* targets and other rod-specific genes, such as *Rho, G protein subunit alpha transducin 1* (*Gnat1*), *Cyclic nucleotide gated channel subunit beta 1* (*Cngb1*)*, Cyclic nucleotide gated channel subunit alpha 1* (*Cnga1*), *Pde6b* and *Nr2e3* (Figure 1B). However, not all the clones could be efficiently propagated in vitro and, based on this parameter, we chose clone A11, which from now on will be called 661W-A11.

### 2.2. Characterization of 661W-A11 Cells

**Figure 2:**
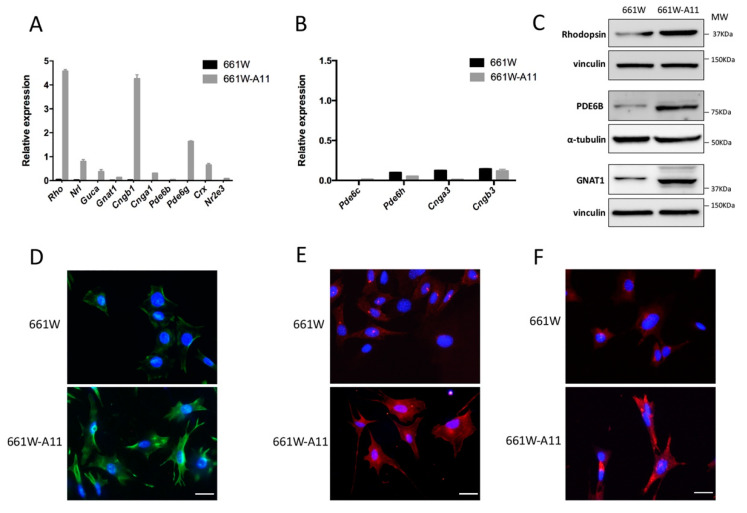
661W-A11 cells express rod-specific genes and proteins. (**A**) Rod-specific mRNAs and (**B**) cone-specific mRNAs were analyzed by real-time qPCR. *S26* was used as a reference gene. (**C**) Immunobloting of rhodopsin, PDE6B and GNAT1 on 661W and 661W-A11 cells. Normalization with vinculin for rhodopsin and GNAT1 and with α-tubulin for PDE6B is shown below each analyzed protein. (**D**) Immunofluorescence analysis of rhodopsin (green) protein on 661W and 661W-A11 cells. (**E**) Immunofluorescence analysis of PDE6B (red) protein on 661W and 661W-A11 cells. (**F**) Immunofluorescence analysis of GNAT1 (red) protein on 661W and 661W-A11 cells. Nuclei were stained with 4′,6-diamidino-2-phenylindole (DAPI): blue. Scale bar: 10 μm.

To further characterize the 661W-A11 clone, expression levels of several rod-specific genes and cone-specific genes were compared by real-time qPCR in the 661W-A11 and 661W cells (Figure 2A,B). We observed that 661W-A11 cells had a significant upregulation of rod-specific gene expression but a similar expression of cone-specific genes, compared with 661W cells. Immunobloting and immunofluorescence confirmed at protein level that expression levels of rhodopsin, PDE6B and GNAT1 were increased in 661W-A11 compared with 661W cells (Figure 2C–F). The 661W-A11 cells displayed a more elongated morphology (Figure 2D–F) and had a slower replication rate compared with 661W. Based on this evidence, we confirmed that clone 661W-A11 acquired rod photoreceptor features rather than cone photoreceptor features.

### 2.3. Mimicking Photoreceptor Degeneration In Vitro

**Figure 3:**
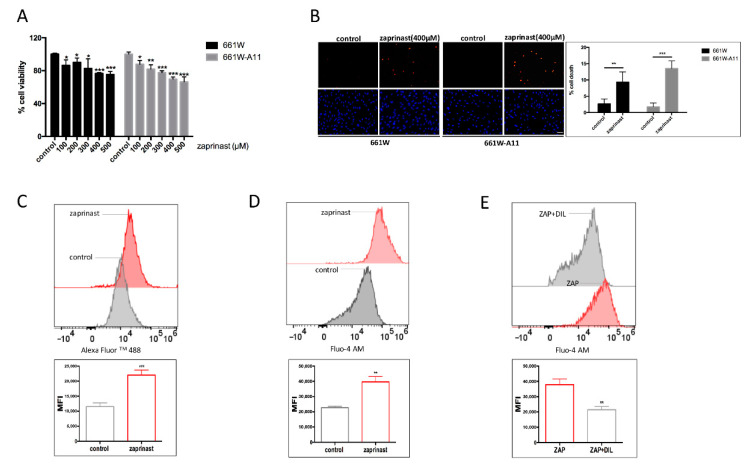
Effect of PDE6B inhibition by zaprinast treatment. (**A**) Cell viability assay, based on MTT, after treatment with scaling concentrations of zaprinast on 661W and 661W-A11 cells. Values of cells treated with the vehicle (0 μM, control) were set as 100% cell viability. (**B**) TUNEL assay detected dying cells (upper panels, red) in zaprinast-treated 661W and 661W-A11 cells; nuclei were stained with DAPI (lower panels, blue). Control shows cells treated with the vehicle (DMSO). Scale bar: 40 μm. On the right-hand side, quantifications of the TUNEL^+^ cells are reported. (**C**) Increased cGMP levels were detected by flow cytometry using an antibody (anti-cGMP) and a secondary antibody labeled with Alexa Fluor 488 in zaprinast-treated versus untreated 661W-A11 cells. Control: cells treated with the vehicle (DMSO); zaprinast: cells treated with 400 μM zaprinast for 24 h. (**D**) Increased Ca^2+^ levels were detected by flow cytometry staining with Fluo-4 AM in zaprinast-treated versus untreated 661W-A11 cells. Control: cells treated with the vehicle (DMSO); zaprinast: cells treated with 400 μM zaprinast for 24 h. (**E**) Decreased Ca^2+^ levels were detected by flow cytometry in zaprinast (+)-*cis* diltiazem-treated versus zaprinast-treated 661W-A11 cells. ZAP: cells treated with 400 μM zaprinast for 24 h; ZAP+DIL: cells pretreated with 50 μM (+)-*cis* diltiazem for 2 h and then treated with 400 μM zaprinast and 50 μM (+)-*cis* diltiazem for 24 h; MFI: mean fluorescence intensity. Statistical comparison: Student’s unpaired two-tailed *t*-test; * *p* < 0.05, ** *p* < 0.01, *** *p* < 0.001.

PDE6 loss of function mutations are found in patients affected by a recessive form of RP (RP40) [33], and this mutation causes an intracellular increase in cGMP, an influx of calcium in photoreceptors and activation of protein kinase G (PKG) and calpains [3]. In order to mimic the photoreceptor degeneration in 661W-A11 cells, a protocol based on treatment with zaprinast was developed. Zaprinast is an inhibitor of PDEs, specifically PDE6 and, to a lesser extent, PDE5 [34]. Scaling concentrations of zaprinast, starting from 100 μM to 500 μM, were applied to 661W and 661W-A11 cells. A dose response of dropped cell viability was observed in both cell types, with around 25% viability reduction detected on 661W and 30% on 661W-A11 when a concentration of either 400 μM or 500 μM was used (Figure 3A); 400 μM was chosen for cell death detection. We determined that exposure to zaprinast for 24 h induced 9% cell death on 661W cells and 14% cell death on 661W-A11 cells, based on a TUNEL assay (Figure 3B).

To demonstrate that this condition could mimic the PDE6 loss of function in 661W-A11 cells, as found in retinal degeneration, we evaluated changes in cGMP and Ca^2+^ levels. cGMP was analyzed by flow cytometry using an antibody specifically binding cGMP, and intracellular calcium was assessed by Fluo-4 AM staining. The effect of zaprinast on blocking the PDE6 enzyme was demonstrated by the statistically significant increase in cGMP and in intracellular calcium (Figure 3C,D).

With the aim of determining the mechanism underlying calcium influx, we exposed zaprinast-treated 661W-A11 to a blocker of L-type voltage gated calcium channels, (+)-*cis* diltiazem. We determined that (+)-*cis* diltiazem could interfere with the zaprinast-induced calcium influx (Figure 3E).

**Figure 4:**
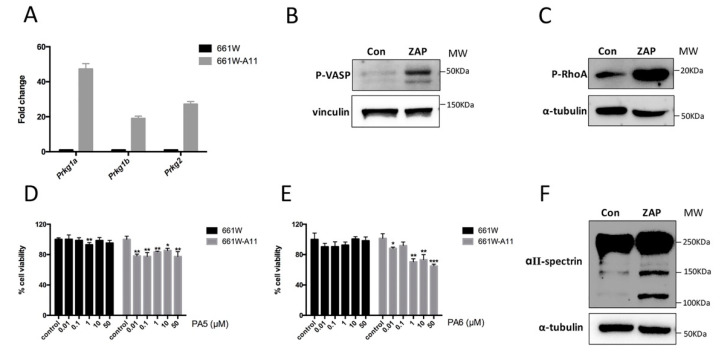
Activation of PKG and calpain proteases in 661W-A11. (**A**) Real-time qPCR analysis of *Prkg1a*, *Prkg1b* and *Prkg2* mRNA in 661W-A11 cells compared with 661W cells. (**B**,**C**) Immunoblotting of phosphorylated VASP (P-VASP) and phosphorylated RhoA (P-RhoA) on control (Con: vehicle-treated) and zaprinast-treated (ZAP) 661W-A11 cells. (**D**,**E**) Cell viability assay, based on MTT, after treatment with increasing doses of PA5 (**D**) and PA6 (**E**). Values of 661W and 661W-A11 cells treated with the vehicle (H_2_O) were set as 100% cell viability. (**F**) Western blot analysis of the effects of αII-spectrin on the control and zaprinast-treated groups shows an increase in the 145–150 kDa fragments resulting from calpain cleavage and of the 120 kDa fragment resulting from caspase cleavage. Con: cells treated with DMSO; ZAP: cells treated with 400 μM zaprinast for 24 h. Statistical comparison: Student’s unpaired two-tailed *t*-test; * *p* < 0.05, ** *p* < 0.01, *** *p* < 0.001.

To investigate activation of PKG in this in vitro model, we first analyzed the gene expression of different PKG isoforms, and found higher mRNA levels of *Prkg1a*, *Prkg1b* and *Prkg2* genes, encoding for the isoforms PKG1α, PKG1β and PKG2, respectively, in 661W-A11 when compared with 661W cells (Figure 4A). PKG activation was evaluated by analyzing the phosphorylation status of two different targets: phosphorylation of the vasodilator-stimulated phosphoprotein (VASP) at Serine 239 and phosphorylation of the Ras homolog family member A (RhoA) at Serine 188. VASP is a protein associated with the cytoskeleton and Serine 239 is one of the main PKG-dependent phosphorylation sites in VASP [35]. Immunoblotting showed that phosphorylated VASP and phosphorylated RhoA were readily detectable in the zaprinast-treated 661W-A11 cells, indicating activation of PKG (Figure 4B,C).

These results suggested that increased cGMP could activate PKG but, in order to determine if zaprinast treatment could be a valuable model for retinal degeneration, we assessed if activation of PKG was correlated with cell death, similar to what can be found in the degenerating retina [36]. To this end, we treated the 661W-A11 as well as 661W cells with increasing concentrations of PA5 and PA6, two cGMP analogs that we previously published as PKG activators [37]. These PKG analogs present different activation constants for the different PKG isoforms, i.e., PA5 targets mostly PKG2 rather than PKG1, and PA6 strongly activates PKG1α and PKG1β rather than PKG2 [37]. We found that only the 661W-A11 cells were sensitive to both activators (Figure 4D,E), confirming that triggering PKG activity is a toxic event for rod-like cells such as 661W-A11 cells.

Previous studies correlated increased intracellular calcium with calpain protease activity [38,39,40]. The activation of calpain proteases was thus evaluated by assessing the cleavage of αII-spectrin, a substrate for calpains. An increased amount of the 145–150 kDa fragments of αII-spectrin derived from calpain cleavage was observed in 661W-A11 cells after zaprinast treatment (Figure 4F).

### 2.4. Validation of the In Vitro Model for Drug Screening

Different drugs have been reported to have a neuroprotective effect in retinal degeneration models [41]. Recombinant human pigmented epithelium-derived factor (PEDF) protein was shown to decrease intracellular calcium and photoreceptor cell death in mouse models of retinal degeneration [23,42]. Similarly, calpastatin peptide, which is an endogenous calpain inhibitor, was reported to protect photoreceptors from cell death when administered in vivo [40]. Moreover, the cGMP inhibitor analog CN03 was shown to limit the activity of PKG and CNGC (cyclic nucleotide-gated ion channel), and to protect photoreceptors from degeneration [18].

**Figure 5:**
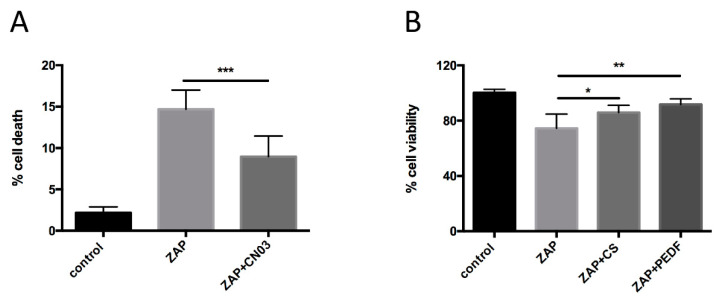
Validation of the in vitro model for drug screening. (**A**) A TUNEL assay was used to determine the percentage of cell death in 661W-A11. (**B**) Cell viability was assessed by MTT assay. The values of cells treated with the vehicle (DMSO, control) were set as 100% cell viability. ZAP: cells treated with 400 μM zaprinast; ZAP+CN03 or CS or PEDF: cells were pretreated with 50 µM CN03 or 20 µM calpastatin peptide (CS) or 10 nM PEDF for 2 h before being stressed with 400 μM zaprinast in the presence of the neuroprotective compounds. Statistical comparison: Student’s unpaired two-tailed *t*-test; * *p* < 0.05, ** *p* < 0.01, *** *p* < 0.001.

The neuroprotective effects of the abovementioned drugs were tested in 661W-A11 cells, and the percentage of cell death or cell viability was analyzed after zaprinast treatment with or without the neuroprotective drugs. The data confirmed that zaprinast treatment increased cell death and decreased cell viability compared with the control. CN03, calpastatin peptide and PEDF confirmed their neuroprotective effects on the newly developed photoreceptor cell model (Figure 5A,B).

## 3. Discussion

A growing number of studies have suggested that the cGMP-dependent activation of intracellular targets drives photoreceptor cell death in a variety of IRD forms. Many mutation-caused retinal dystrophies, including RP and Leber congenital amaurosis (LCA), are known to be associated with cGMP-dependent photoreceptor cell death [6,43]. The dysregulation of cGMP, typically its high levels, may trigger downstream processes that are toxic to photoreceptors [44]. Here, we developed a cell line displaying rod-specific features and showed that upon PDE6 inhibition, which elevated cGMP and calcium levels, cell death pathways previously described in rodent models of RP were activated [3]. While we cannot rule out the possibility that not all the cell death mechanisms were mirrored in this in vitro model, we can confirm that the 661W-A11 cell line could be used as an in vitro model for the study of rod photoreceptor cell death in RP and other IRDs linked to increased intracellular cGMP. This limitation of the in vitro model presented here needs to be pondered when using this system for high-throughput screening.

The link between calcium influx and photoreceptor cell death has already been established in retinal degeneration. It is hypothesized that high levels of cGMP lead to calcium influx through CNGC and that excessive calcium triggers photoreceptor cell death [45]. Two major channels for calcium influx are found in photoreceptors: the CNGC in the photoreceptors’ outer segment, and the voltage-gated calcium channels in the photoreceptor cells’ body [46]. When (+)-*cis* diltiazem, a benzothiazepine drug known to target the L-type voltage gated channel, was applied to 661W-A11 cells, we observed a decrease in zaprinast-induced calcium influx. The neuroprotective effect of (+)-*cis* diltiazem on RP models is quite contradictory: an early study showed that (+)-*cis* diltiazem was able to prevent rod degeneration in a *rd1* mutant mouse, and some studies confirmed these data but other ones were unable to reproduce its protective effects [47,48,49]. In our model, we could observe a reduction in intracellular calcium upon (+)-*cis* diltiazem exposure, suggesting that the calcium entered mainly through the L-type channel. However, (+)-*cis* diltiazem has also a moderate effect on CNGC [50], so we cannot exclude the possibility of calcium influx from CNGC, as we showed the expression of mRNA encoding the CNGC subunits in the cells. We favor the first hypothesis because transport and diffusion of Ca^2+^ between outer segment, harboring the CNGC, and the inner segment, containing the photoreceptor cytoplasm, appears to be limited in the retina. L-type calcium channels are, in fact, the main pathway of Ca^2+^ entrance from the extracellular space in photoreceptor cells [51].

Activation of PKGs plays a role in cell death in several neuronal cell types [52], and studies have demonstrated that cGMP-dependent overactivation of PKG acts as an important cell death mechanism in photoreceptor degeneration [36]. In mammals, two different genes encode PKG: *PRKG1* for the isoforms PKG1α and PKG1β, and *PRKG2* for PKG2 [53]. Their expression in the retina was demonstrated and the correlation with retinal degeneration was shown in a CNGC loss-of-function mouse model in which the knock-out of *Prkg1* led to sustained rod cell survival [54]. PKG expression has been demonstrated in the retina and PKG activity was characterized in 661W cells [55,56,57]. In 661W-A11, we demonstrated increased expression of *Prkg1* and *Prkg2* genes. The key role of PKG in the cell death mechanism was confirmed with PKG stimulation, which was demonstrated to be detrimental to the cells, and by PKG activation upon intracellular cGMP increase. Interestingly, 661W-A11 cells exhibited low tolerance to PA5, which preferentially activates PKG2. PA6, which acts preferentially on PKG1, was less effective. Several studies have shown that specific activation of PKG2 interferes with proliferation and triggers pro-apoptotic effects in cancer cell lines [58,59,60,61]. Until now, not much has been known about the role of different PKG isoforms in photoreceptors; thus the identification of PKG substrates and their downstream pathways would significantly accelerate the identification of new targets in IRD drug development.

In summary, 661W-A11 cells may serve as an alternative cell model for studying the cGMP-dependent PKG activation in photoreceptor cell death and for high-throughput drug screening.

## 4. Materials and Methods

### 4.1. Cell Culture, Genetic Modification and Treatments

661W cells were cultured in low glucose (1 mg/mL) DMEM (Dulbecco’s Modified Eagle Medium, 22320022 Gibco) supplemented with 10% fetal bovine serum (FBS), 2 mM glutamine, 100 U/mL penicillin and 100 µg/mL streptomycin, purchased from Thermo Fisher Scientific (Rodano, Italy).

Isolation of single-cell clones: 661W cells were transduced with a replication-incompetent retrovirus expressing NRL. The retrovirus was generated by cloning murine *Nrl* cDNA (accession number: L14935) between the *Eco*RI and *Xho*I restriction sites of the pLXSN vector for the production of a retrovirus also expressing the neomycin resistance gene. Transduced cells were selected by treatment with G418, and clones were generated by limiting dilution. RNA extracted from single-cell clones was retrotranscribed and *Nrl* expression was evaluated by RT-PCR (see below). 

Next, 20,000 cells were seeded onto glass coverslips coated with 3 μg/mL laminin in 24-well plates or 6000 cells per well in 96-well plates. The following day, cells were treated for 2 h with a medium containing either 10 nM PEDF (a kind gift from S.P. Becerra) or 20 μM calpastatin peptide (208902 Calbiochem) or 50 μM CN03 (P 007 Biolog, Bremen, Germany) or 50 µM (+)-*cis*-diltiazem (D2521 Sigma, Milan, Italy), and then stressed with 400 μM zaprinast (Z0878 Sigma) for 24 h, or by adding an equal volume of DMSO as a control. For PA5 and PA6 treatments, cells were treated with different drug concentrations for 24 h or by adding an equal volume H_2_O as a control.

### 4.2. RT-PCR and Real-Time qPCR

Total RNA was extracted from cells using the RNeasy Mini Kit (74104 Qiagen, Milan, Italy) according to the manufacturer’s instructions. cDNA was synthesized from 3 µg of RNA using the Transcriptor High Fidelity cDNA Synthesis Kit (04379012001 Roche). PCR was performed with 1 μL cDNA using specific primers (Table 1) under the following conditions: 30 s at 94 °C, 30 s at 60 °C and 30 s at 72 °C for 35 cycles. Amplified products were analyzed by electrophoresis in a 1% agarose gel and visualized by ethidium bromide staining. Real-time qPCR was carried out with the Universal SYBR Green Supermix (1725120 Bio-Rad, Segrate, Italy) using 1 µL cDNA on the CFX96 real-time PCR detection system (Bio-Rad) under the following conditions: 30 s at 95 °C, and 39 cycles of 5 s at 95 °C and 30 s at 60 °C; the relative gene expression level was calculated using the ribosomal protein gene *RPS26* as a reference. The primers used for quantitative PCR are listed in Table 1.

### 4.3. Immunofluorescence and TUNEL Assay

Cells were seeded onto glass coverslips in a 24-well plate at a density of 2 × 10^4^ cells/well. After incubation, cells were rinsed with phosphate-buffered saline (PBS) and fixed with 2% paraformaldehyde (PFA) for 10 min. For immunofluorescence, cells were blocked and permeabilized with 3% bovine serum albumin (BSA) and 0.1% Triton X-100 in PBS for 1 h at room temperature. After incubation with primary antibodies in PBS overnight at 4 °C, cells were washed 5 times with PBS and incubated with secondary antibodies for 1 h at room temperature. For nuclear staining, cells were stained with 0.1 μg/mL DAPI. Slides were mounted with Mowiol 4–88 and observed using the Zeiss Axio Imager A2 fluorescence microscope. The primary antibodies used were as follows: anti-rhodopsin (1D4, MAB5356 Sigma, 1:100), anti-PDE6B (ab5663 Abcam, Cambridge, UK 1:100) and anti-GNAT1 (SAB4501223 Sigma, 1:300). Secondary antibodies were used as follows: anti-mouse Alexa Fluor 488 (A11001 ThermoFisher, Rodano, Italy, 1:1000), anti-rabbit Alexa Fluor 568 (A11011 ThermoFisher, 1:1000). Cell death was detected by a TUNEL assay using the In Situ Cell Death Detection Kit, TMR red (12156792910 Roche, Monza, Italy), according to the manufacturer’s instructions. For each replicate, images of 10 different fields were acquired (139.51 µm × 105.21 µm), and dead cell quantification was performed by counting all TUNEL-labeled cells in each field and dividing this by the total number of cells, based on the nuclear DAPI staining. The average cell death percentage from at least 3 biological replicates was plotted using GraphPad Prism.

### 4.4. Flow Cytometry Analysis of cGMP and Calcium

Cells were detached, collected by centrifugation at 300× *g* for 5 min and fixed with cold 2% PFA at room temperature for 20 min. After washing with PBS and centrifugation, cells were resuspended in a permeabilization buffer (0.1%Triton X-100 in PBS) and incubate for 20 min at room temperature. After centrifugation, cells were incubated with a blocking buffer (3% BSA in PBS) for 30 min. Cells were then incubated with the cGMP primary antibody (Jan de Vente and Harry Steinbusch, Maastricht University, The Netherlands, 1:500) for 30 min at room temperature. After rinsing the cells 3 times with PBS, cells were resuspended in the anti-sheep Alexa Fluor 488 secondary antibody (A11015 ThermoFisher, 1:1000) and incubated for 30 min at room temperature. Cells were washed 3 times with PBS and resuspended in 500 μL of PBS. For the analysis of intracellular calcium levels, cells were incubated with 1 μM Fluo-4 AM (F14201 ThermoFisher) at 37 °C for 30 min in Hank’s Balanced Salt solution (HBSS, 14170112 ThermoFisher) in the dark. After incubation, cells were washed twice with HBSS and once with PBS. Cells were detached by treatment with Accutase (SCR005 Millipore, Milano, Italy), collected by centrifugation at 300× *g* for 5 min at room temperature and resuspended in 500 μL of PBS. Cells were immediately analyzed using the Attune NxT Acoustic Focusing Cytometer. Fluorescence was measured at an excitation wavelength of 488 nm; mean fluorescence intensity and fluorescence-positive cells were calculated from 3 biological replicates. Throughout the whole analysis, channel gain and voltage were maintained unvaried.

### 4.5. Western Blotting

For detection of rhodopsin, PDE6B and GNAT1, cells were lysed with a buffer containing 1× PBS, 17mM CHAPS and 1% protease inhibitor. Equivalent amounts of protein extracts (40 μg) were resolved by SDS-PAGE, and immunoblotting was performed following standard procedures. For detection of P-VASP, P-RhoA and αII-spectrin, cells were lysed in a lysis buffer (50 mM Tris·HCl (pH 7.5), 50 mM NaCl, 1 mM EDTA, 5 mM NaH_2_PO_4_, 1 mM DTT, 1% protease inhibitor and 1% phosphatase inhibitor). Equivalent amounts of protein extracts (20 μg) were resolved by SDS-PAGE, and immunoblotting was performed following standard procedures. The antibodies used for Western blotting were as follows: anti-rhodopsin (1D4, MAB5356 Sigma, 1:1000), anti-PDE6B (ab5663 Abcam, 1:500), anti-GNAT1 (SAB4501223 Sigma, 1:1000), anti-vinculin (V4139 Sigma, 1:10,000), anti-α-tubulin (T6199 Sigma, 1:2000), anti-αII-spectrin (AA6, BML-FG6090 Enzo Life, 1:2000), anti-P-VASP (S239, 0047-100/VASP-16C2 Nanotools 1:2000) and anti-P-RhoA (ser188) (S188, ab32046 Abcam 1:1000). The detection was based on a chemiluminescent substrate (Clarity Max Western ECL Substrate 170-5062 Bio-Rad) and images were acquired by a ChemiDoc Touch Imaging System (Bio-Rad, Segrate, Italy).

### 4.6. MTT Assay

Cells were cultured on a 96-well plate at a density of 6000 cells/well. After treatment with the corresponding drugs, the medium was aspirated and the cells in each well were incubated with 50 μL of 1 mg/mL of 3-(4,5-dimethylthiazol-2-yl)-2,5-diphenyl tetrazolium bromide (MTT) in the culture medium for 90 min at 37 °C. After supernatant removal, the purple formazan crystals were dissolved in 100 μL of isopropanol. The plate was shaken for 10 min and optical density (OD) was measured at 570 nm using a microplate reader (Labsystems Multiskan MCC/340, Fisher Scientific, Rodano, Italy). The average absorbance from at least 3 biological replicates was plotted using GraphPad Prism.

### 4.7. Statistical Analysis

Data from each experiment, obtained from at least 3 biological replicates, were presented as means ± SD. Statistical analysis was performed with GraphPad Prism version 7 (GraphPad software, San Diego, CA, USA), and each dataset was analyzed by an unpaired Student’s *t*-test. A value of *p* < 0.05 was considered significant.

## Figures and Tables

**Table 1:** Primers used for RT-PCR and real-time PCR.

	Forward Primer	Reverse Primer
*RPS26*	5′-AAGTTTGTCATTCGGAACATT-3′	5′-GATCGATTCCTAACAACCTTG-3′
*Rho*	5′-AATCTCGAGGGCTTCTTTGC-3′	5′-CCACGTAGCGCTCAATGGC-3′
*Nrl*	5′-GCTACTATTCAGGGAGCCC-3′	5′-GCAGCTGCCGGTTCA-3′
*Gnat1*	5′-GAGCCTCAGAATACCAGCTC-3′	5′-GGCACATATCCTGGAGTCAC-3′
*Cngb1*	5′-TCTGGCTCCTCATGGATTAC-3′	5′-TGATGTCCCCGCCTTTGAC-3′
*Cnga1*	5′-CAACTGGACGATGATTATTGC-3′	5′-TCACTAGCAGCCCTT-3′
*Pde6b*	5′-GGAGAGGACTGTCTTGGATC-3′	5′-GAGCTCAGCTGCTTTGTTCC-3′
*Nr2e3*	5′-TCCCACAGAGTTTGCCTGC-3′	5′-CTCCACGTGCTCAGGATCCT-3′
*Nr2e3* *	5′-GAAACACGAGGCCTGAAGGA-3′	5′-GGGAGCAGGAGGAGCAATTT-3′
*Guca1a*	5′-TGCATAGACAGGGACGAGC-3′	5′-GCACTCATGGATGAGTCGC-3′
*Pde6g*	5′-ATCCCTGGAATGGAAGGCC-3′	5′-TAAATGATGCCATACTGGGC-3′
*Crx*	5′-TAAGATCAATCTGCCTGAGTC-3′	5′-GCTGTTGCTGTTTCTGCTGC-3′
*Pde6c*	5′-GTGGAGTCCCGGAGAAGC-3′	5′-GTCCTGATGGTGTACAGTGC-3′
*Pde6h*	5′-CAGTTCAAGAGCAAGCCTCC-3′	5′-TTCCCAGGGACAGATGACC-3′
*Cnga3*	5′-GCTGGTTCGAGCCCGGAC-3′	5′-CCAGCTTGAAGTGCAAGGTC-3′
*Cngb3*	5′-TGGAAGCCAGCTCTCAGAC-3′	5′-CTCTGGGGTTTGAAAGAAAAC-3′
*Prkg1a*	5′-GCGTTCCGGAAGTTCACTAA-3′	5′-GCCACAATCTCCTGGATCTG-3′
*Prkg1b*	5′-CTTCTACCCCAAGAGCCCAC-3′	5′-ACAATCTCCTGGATCTGTGACAG-3′
*Prkg2*	5′-CCTGACATTTCATCCGGAGG-3′	5′-TTCCGTCACCTTTACGGAGAG-3′

* Primers used only for real-time qPCR.

## Data Availability

Supporting results can be requested from the corresponding author; they are currently stored in a laboratory-owned archive, which will be made open later.
